# The reduction in maize leaf growth under mild drought affects the transition between cell division and cell expansion and cannot be restored by elevated gibberellic acid levels

**DOI:** 10.1111/pbi.12801

**Published:** 2017-09-04

**Authors:** Hilde Nelissen, Xiao‐Huan Sun, Bart Rymen, Yusuke Jikumaru, Mikko Kojima, Yumiko Takebayashi, Rafael Abbeloos, Kirin Demuynck, Veronique Storme, Marnik Vuylsteke, Jolien De Block, Dorota Herman, Frederik Coppens, Steven Maere, Yuji Kamiya, Hitoshi Sakakibara, Gerrit T.S. Beemster, Dirk Inzé

**Affiliations:** ^1^ Department of Plant Biotechnology and Bioinformatics Ghent University Gent Belgium; ^2^ Center for Plant Systems Biology VIB Gent Belgium; ^3^ Growth Regulation Research Group Plant Science Center RIKEN Yokohama Japan; ^4^ Plant Productivity Systems Research Group Plant Science Center RIKEN Yokohama Japan; ^5^ Department of Biology University of Antwerp Antwerp Belgium

**Keywords:** maize, mild drought, gibberellic acid, cell division

## Abstract

Growth is characterized by the interplay between cell division and cell expansion, two processes that occur separated along the growth zone at the maize leaf. To gain further insight into the transition between cell division and cell expansion, conditions were investigated in which the position of this transition zone was positively or negatively affected. High levels of gibberellic acid (GA) in plants overexpressing the GA biosynthesis gene *GA20‐OXIDASE* (*GA20OX‐1* ^*OE*^) shifted the transition zone more distally, whereas mild drought, which is associated with lowered GA biosynthesis, resulted in a more basal positioning. However, the increased levels of GA in the *GA20OX‐1* ^*OE*^ line were insufficient to convey tolerance to the mild drought treatment, indicating that another mechanism in addition to lowered GA levels is restricting growth during drought. Transcriptome analysis with high spatial resolution indicated that mild drought specifically induces a reprogramming of transcriptional regulation in the division zone. ‘Leaf Growth Viewer’ was developed as an online searchable tool containing the high‐resolution data.

## Introduction

Plants are continuously producing organs that grow to fulfil specific roles during plant development, and the sessile nature of plants urges them to adjust their growth when the environment changes. Therefore, it is pivotal to gain better insights into the coordination of the processes of cell division and cell expansion and to understand how intrinsic signals and environmental cues impinge on these. Depending on the organ, the processes of cell division and cell expansion are mainly viewed as spatially and/or temporally regulated (Gonzalez *et al*., 2012; Nelissen *et al*., 2012; Sozzani and Iyer‐Pascuzzi, 2014), and it becomes increasingly clear that the regulation of the growth mechanisms in dicot and monocot leaves is to a great extend conserved (Nelissen *et al*., 2016).

One of these conserved mechanisms that also represents an important developmental switch is the transition from cell division to cell expansion, for which already a large number of genes and molecular pathways have been identified (Breuninger and Lenhard, 2010; Gonzalez *et al*., 2012; Nelissen *et al*., 2012; Sozzani and Iyer‐Pascuzzi, 2014). These genes function in transcriptional regulation (Gonzalez *et al*., 2010; Horiguchi *et al*., 2005; Mizukami and Fischer, 2000; Nath *et al*., 2003; Vercruyssen *et al*., 2014), protein degradation (Disch *et al*., 2006; Li *et al*., 2008), hormone metabolism and signalling (Achard *et al*., 2009; Hu *et al*., 2003) and the production of a non‐cell‐autonomous growth‐promoting signal (Czesnick and Lenhard, 2015; Kazama *et al*., 2010). In Arabidopsis, the cell cycle arrest front, visualized by a *CYCLINB1;1* reporter gene (Donnelly *et al*., 1999), is often used together with the analysis of growth over time (kinematic analysis; Andriankaja *et al*., 2012; Nelissen *et al*., 2013) to gain insights into perturbations in the transition from cell division to cell expansion. Differences in the cell cycle arrest front have been observed in genetic (Mizukami and Fischer, 2000; Nath *et al*., 2003; Vercruyssen *et al*., 2014) and environmental (Skirycz *et al*., 2011) perturbations.

During steady‐state growth of the maize leaf, the growth‐promoting processes are spatially separated. At the leaf base, cells are dividing, and as the cells move upwards in the leaf, they transition to expanding and later to mature cells (Nelissen *et al*., 2013). The regions in the maize growth zone in which these processes occur, are quite large, encompassing more than 10 mm and are referred to as division zone (DZ), expansion zone (EZ) and mature zone (MZ). The respective transitions are defined here as transition zone 1 (TZ1) and transition zone 2 (TZ2). TZ1 has been shown to be characterized by a local accumulation of bioactive gibberellic acid (GA; Nelissen *et al*., 2012). This GA peak is instrumental to determine the position of TZ1, and thus in defining the position where cells decide to exit cell division and to enter cell expansion (Nelissen *et al*., 2012). Water deficit has been shown to affect the growth‐promoting processes differently depending on the position along the growth zone (Tardieu *et al*., 2011, 2000).

The aim of this study was to gain further insights into the molecular processes that dynamically regulate the transition from cell division to cell expansion in growing maize leaves. Therefore, we examined the effects of perturbations that positively (overexpression of *GA20OX‐1*; Nelissen *et al*., 2012) or negatively (mild drought stress) affect the position of TZ1. Strikingly, a reduced level of bioactive GA (GA_1_ and GA_4_) and its biosynthetic precursors was observed at TZ1 under mild drought conditions. However, merely increasing GA levels was insufficient to overcome this mild drought phenotype, because the *GA20OX‐1* ^*OE*^ line was not more drought tolerant, compared with its nontransgenic siblings. We analysed the high‐resolution transcriptomics data of mild drought‐treated and *GA20OX‐1* ^*OE*^ plants by aligning the samples according to the relative position of TZ1. In this manner, growth zone‐specific, mild drought‐induced changes in the expression of transcription factors, E2F/DP target genes, aquaporins and photosynthesis‐related genes were shown. To visualize and provide access to these high‐resolution data, we developed ‘Leaf Growth Viewer’ (LGV).

## Results

### Mild drought affects the position of transition zone 1

Overexpression of the rate‐limiting GA biosynthesis gene *GA20‐OXIDASE* (*GA20OX‐1* ^*OE*^) has an enhancing effect on the size of the DZ and results in a more distal position of TZ1 (Nelissen *et al*., 2012). Here, we investigated the effect of mild drought on this transition zone between cell division and cell expansion in B104, an inbred that is closely related to B73 (Liu *et al*., 2003) and that can be routinely transformed (Coussens *et al*., 2012; Frame *et al*., 2006).

Our mild drought conditions reduced the leaf elongation rate (LER) by 28% compared with plants grown in well‐watered conditions (Table 1). A kinematic analysis revealed that this growth reduction was partially the result of a reduced cell production (16%), which was in turn caused by a reduction in DZ size (22%, *P*‐value = 0.04), bringing about a significantly reduced cell number (14%, *P*‐value = 0.005), and thus a more basal position of TZ1. The duration of one cell cycle and the rate of cell division were not significantly altered (*P*‐value = 0.5 and 0.4, respectively, Table 1). In addition to this effect on cell division, also a reduction in cell expansion was observed, because the mature cell size was significantly lowered by 15% (*P*‐value = 0.04). Whereas both cell division and cell expansion were negatively affected by mild drought, the final reduction in leaf length was remarkably less pronounced (10%, *P*‐value = 0.002), because the duration of growth, also referred to as leaf elongation duration (LED), was significantly increased by 14% (*P*‐value = 0.0025), indicating that although under mild drought the LER was reduced, growth was maintained for a longer period of time. The same mild drought treatment resulted in a similar repositioning of TZ1 and an increased LED in B73 (Table 1).

**Table 1:** Growth‐related parameters of mild drought‐treated and well‐watered B104 and B73 plants

Parameters	B104				B73			
	Control*	Drought*	% Change	*P*‐value#	Control*	Drought*	% Change	*P*‐value#
LER (mm/h)	2.9 ± 0.1	2.1 ± 0.1	−29	8.10^−5^	3.1 ± 0.1	2.2 ± 0.2	−30	0.008
FLL (mm)	557 ± 8.6	499.2 ± 9.8	−10	0.002	na	na	na	na
Lma (μm)	123 ± 1	104 ± 1	−15	0.04	136 ± 7	110 ± 4	−15	0.07
Lez (mm)	28.6 ± 2.3	26.7 ± 2.4	−7	0.6	50.0 ± 0.8	47.0 ± 2.6	−7	0.3
Nez	393 ± 32	469 ± 56	19	0.3	657 ± 71	882 ± 142	34	0.2
*P* (cells/h)	24 ± 0.23	20 ± 0.2	−16	7.10^−4^	23 ± 1	19 ± 1	−18	0.05
Ldz (mm)	11.6 ± 0.6	9 ± 0.2	−22	0.04	14.9 ± 1.5	9.8 ± 0.4	−34	0.03
Ndz (cells)	613 ± 11	528 ± 7	−14	0.005	747 ± 78	619 ± 9	−17	0.2
*D* (cells/cells.h)	0.039 ± 0.0001	0.038 ± 0.001	−2	0.4	0.032 ± 0.005	0.030 ± 0.002	−4	0.8
Tc (h)	18 ± 0.4	18 ± 0.1	2	0.5	23 ± 3	23 ± 1	2	0.9

^*^Mean ± standard error. ^#^ *P*‐values as obtained by Student's *t*‐test (*n* = 3).

na, not analysed; LER, leaf elongation rate; FLL, final leaf length; Lma, mature cell length; Lez, expansion zone size; Nez, number of cells in expansion zone; *P*, cell proliferation rate; Ldz, division zone size; Ndz, number of dividing cells; *D*, cell division rate; Tc, cell cycle duration.

### Mild drought lowers auxin and cytokinin levels in the division zone and gibberellic acid levels at the transition zone

**Figure 1:**
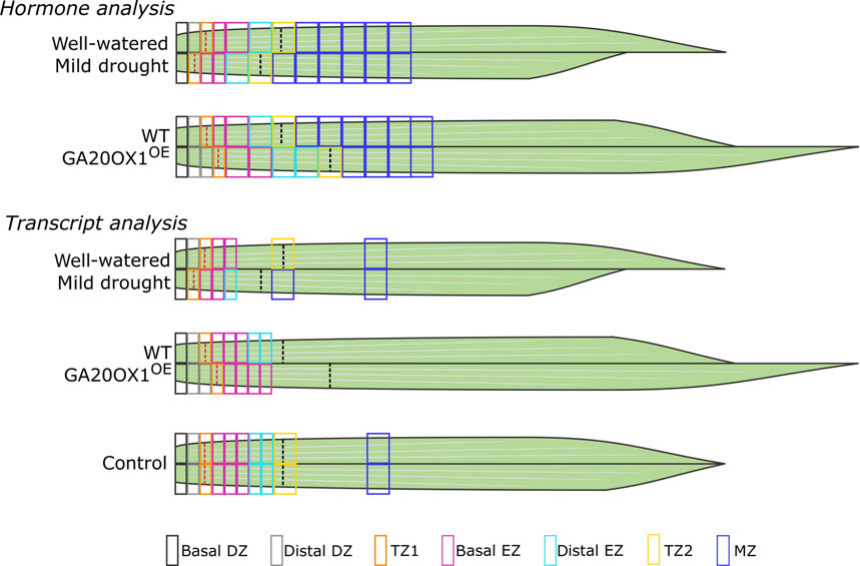
Schematic overview of the sampling strategy of leaf four samples for hormone and transcriptome analysis. The transition between cell division and cell expansion (TZ1) is indicated by a red dotted line, and the transition between cell expansion and mature cells (TZ2) is indicated by a black dotted line. Although the boundaries between the different zones look fixed, the transition between different zones is a gradual process. To investigate the robust transcriptional changes with high resolution in the growth zone, the control samples from the mild drought (well‐watered) and *GA20OX‐1* ^*OE*^ (WT) experiment were analysed together (bottom panel). WT, wild type; DZ, division zone; TZ, transition zone; EZ, expansion zone; MZ, mature zone.

The effect of the repositioning of TZ1 by elevated GA levels or mild drought was assessed at the hormone level. The growth zone was sampled every 0.5 cm, while the remainder of the leaf until 10 cm was sampled in 1‐cm pieces (Figure 1). Because TZ1 in B104 is positioned around 1.3 cm, the most basal half cm reflects the basal DZ, while the second half cm represents the distal DZ. The third half cm coincides with the location of TZ1 and thus contains cells that are both dividing and expanding. The fourth half cm contains cells in early cell expansion, while later phases of cell expansion and cell maturity are present in the successive 1‐cm‐long leaf samples taken along the leaf gradient (Figure 1). TZ2 is located between 4 and 5 cm in B104 plants.

**Figure 2:**
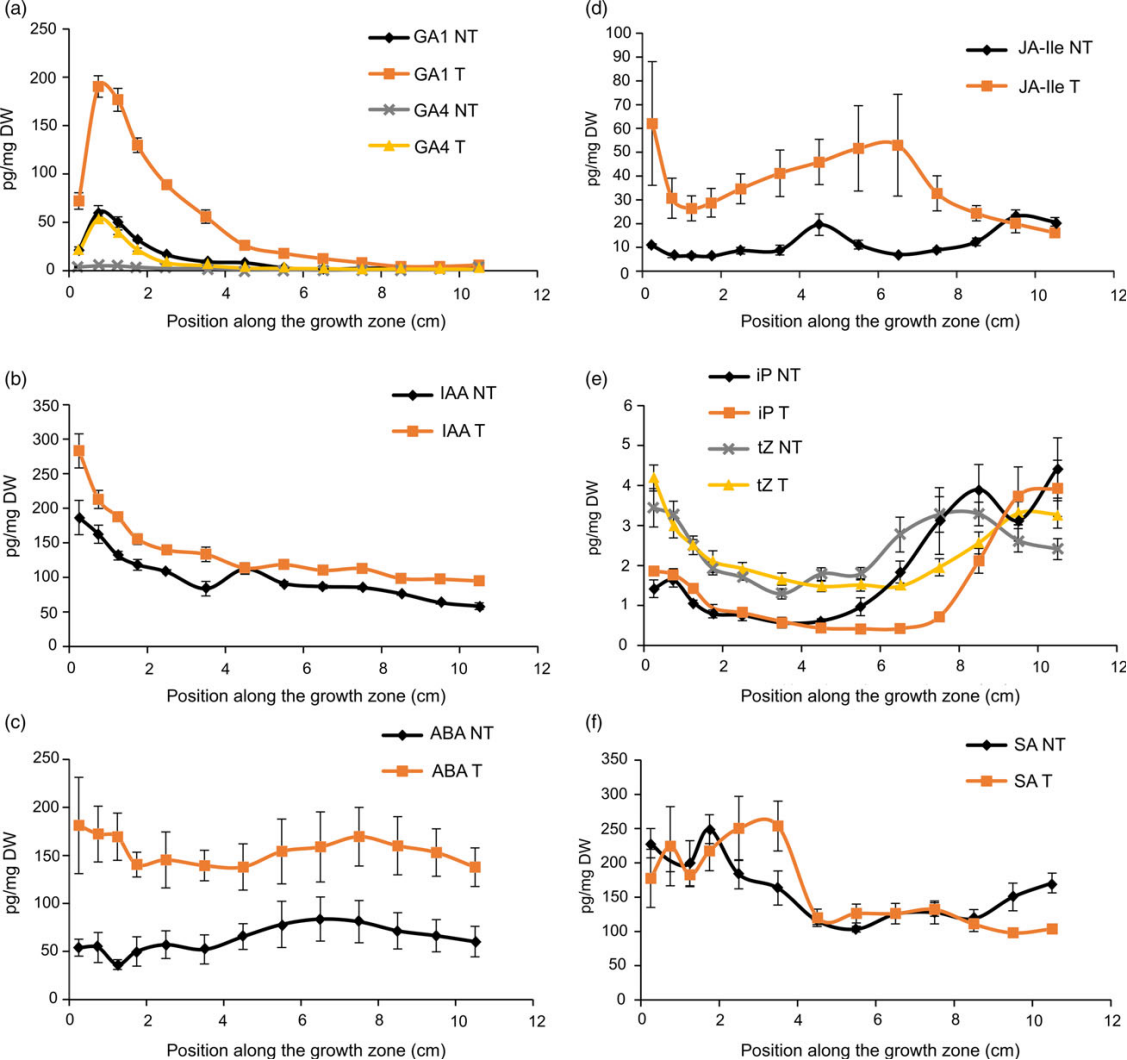
Hormone levels [pg/mg dry weight (DW)] along the growth zone of transgenic *GA20OX‐1* ^*OE*^ plants (T) and segregating nontransgenic siblings (NT): (a) gibberellic acids (GA); (b) auxin indole‐3‐acetic acid (IAA); (c) abscisic acid (ABA); (d) jasmonoyl‐isoleucine (Ja‐Ile); (e) cytokinins N6‐(Δ2‐isopentenyl)adenine (iP) and trans‐zeatin (tZ); (f) salicylic acid (SA). Values are average ± standard error (*n* = 5).

There was a large increase in bioactive GAs (GA_1_ and GA_4_) in *GA20OX‐1* ^*OE*^ plants (Figure 2a), confirming our previous findings (Nelissen *et al*., 2012). The higher GA levels also resulted in elevated levels of the auxin indole‐3‐acetic acid (IAA; Figure 2b), abscisic acid (ABA; Figure 2c) and jasmonoyl‐isoleucine (JA‐Ile; Figure 2d). Cytokinin levels [N^6^‐(Δ^2^‐isopentenyl)adenine (iP) and trans‐zeatin (tZ)] were unaffected in the DZ of the *GA20OX‐1* ^*OE*^ line, but showed a delayed rise towards the end of the EZ (Figure 2e). This rise in iP corresponds to the position of TZ2, which is shifted to a more distal position as compared with the nontransgenic siblings (Figure 1), suggesting that iP plays a role at the transition between cell expansion and cell maturity. Salicylic acid (SA) levels were not significantly affected in the *GA20OX‐1* ^*OE*^ plants (Figure 2f).

**Figure 3:**
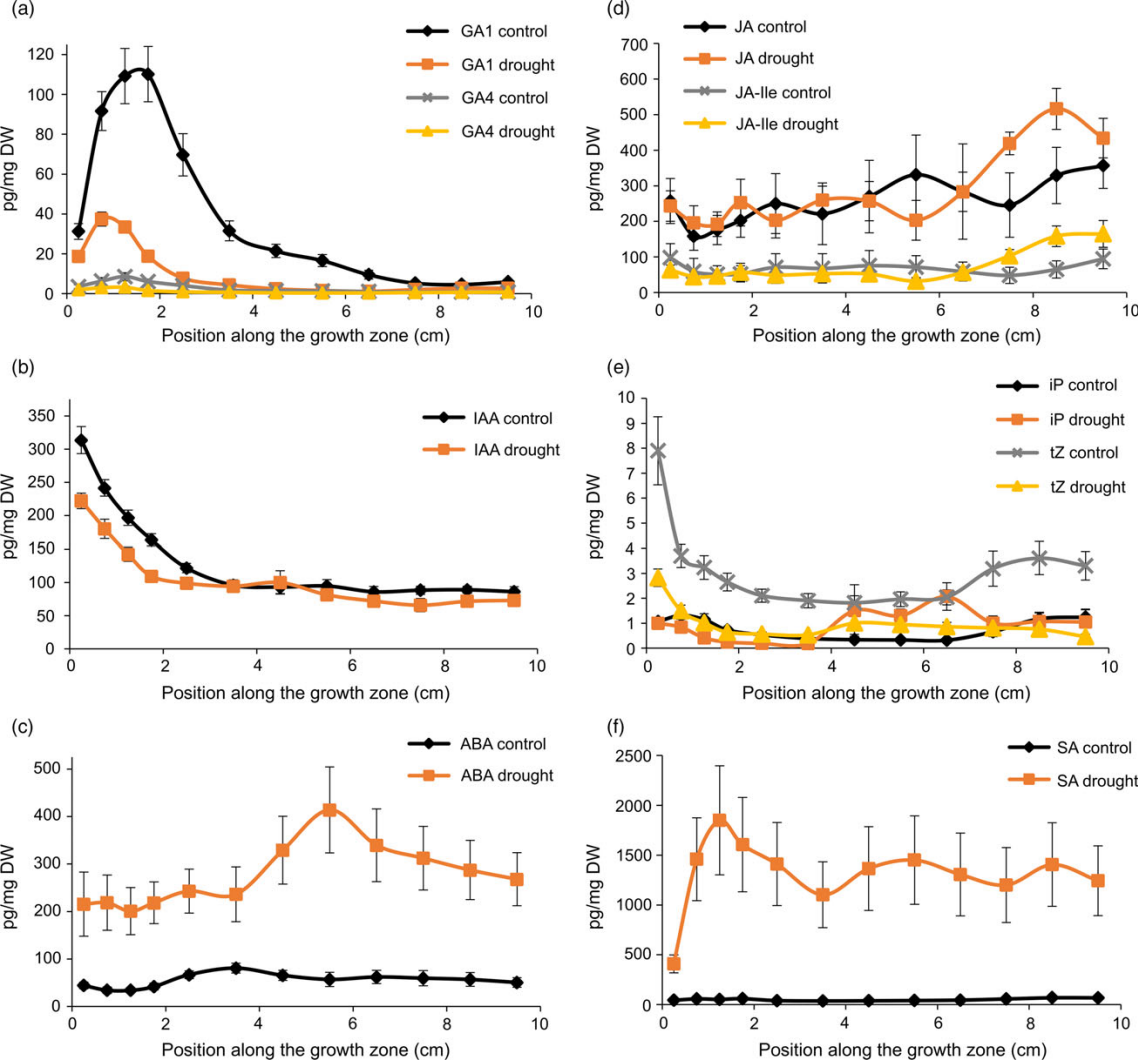
Hormone levels (pg/mg dry weight (DW)) along the growth zone under mild drought and well‐watered (control) conditions: (a) gibberellic acids (GA); (b) auxin indole‐3‐acetic acid (IAA); (c) abscisic acid (ABA); (d) jasmonic acid (JA) and jasmonoyl‐isoleucine (Ja‐Ile); (e) cytokinins N6‐(Δ2‐isopentenyl)adenine (iP) and trans‐zeatin (tZ); (f) salicylic acid (SA). Values are average ± standard error (*n* = 5).

Under mild drought, the levels of ABA and SA (Figure 3c,f) showed a strong increase. Strikingly, the levels of SA under mild drought significantly increased between the basal and distal DZ to stay maximal throughout the EZ and MZ (Figure 3f). No significant differences between mild drought and well‐watered plants were found for JA and JA‐Ile levels (Figure 3d). Under mild drought, both IAA and tZ levels were found to be significantly decreased at those positions in the growth zone where their levels were the highest in well‐watered conditions, that is at the leaf base (Figure 3b,e). In contrast to the more distal shift of the position of TZ2 in the *GA20OX‐1* ^*OE*^ plants, its position (Figure 1) and a concomitant rise in iP (Figure 3e) were shifted to a more basal position under mild drought as compared with the well‐watered B104. The levels of the bioactive GA_1_ and GA_4_ (Figure 3a) were down‐regulated by mild drought and maximal levels were reached approximately 5 mm closer to the leaf basis as compared with control leaves. This shift in GA maxima towards the leaf base corresponds to the shift in TZ1 at the cellular level (Table 1).

### GA biosynthesis is lowered by mild drought stress

**Figure 4:**
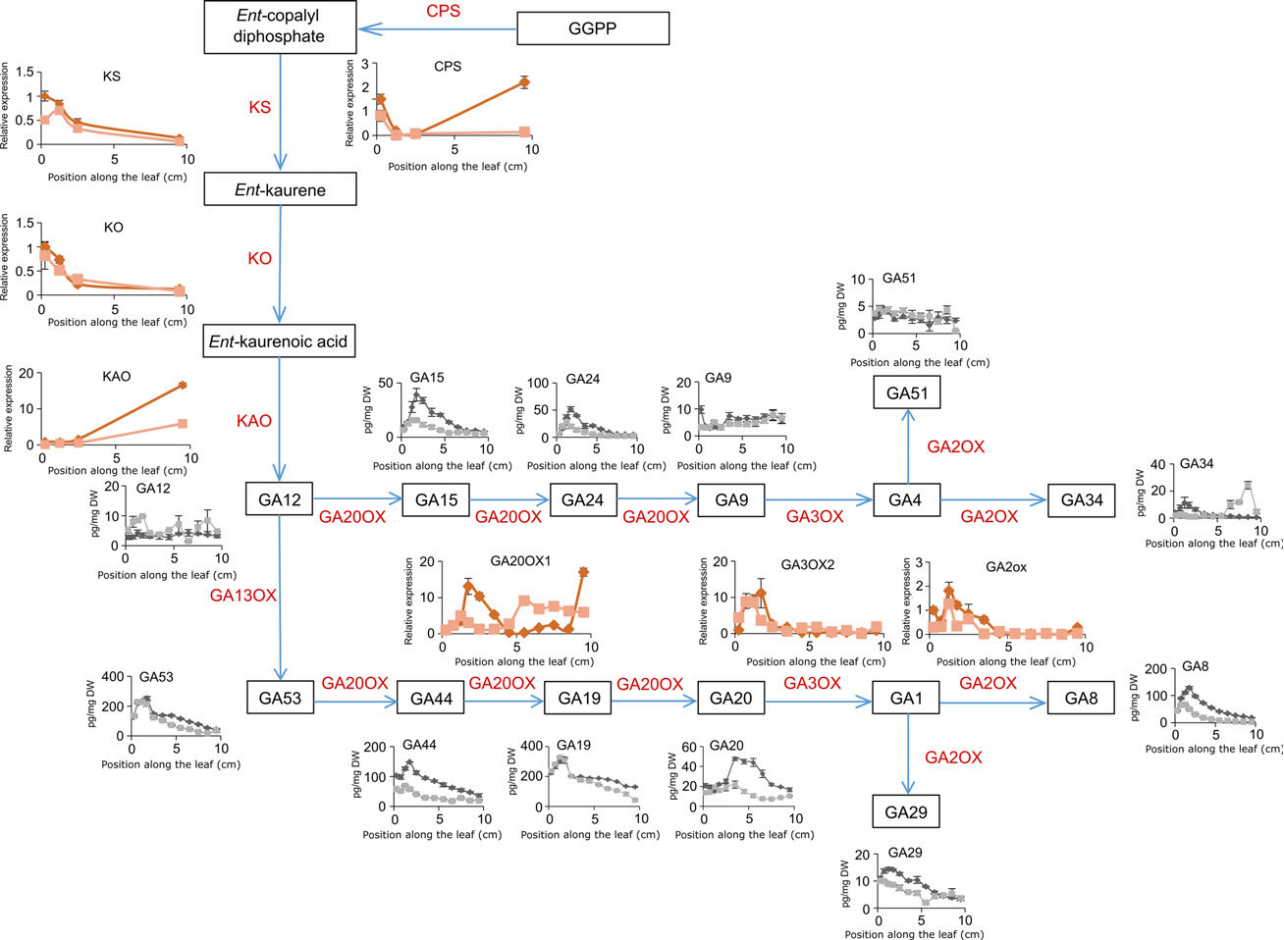
Gibberellic acid (GA) biosynthesis and catabolism along the growth zone of B104 leaves. GA metabolites along the growth zone under mild drought and well‐watered conditions (grey and black, respectively). Values are average ± standard error (*n* = 5). The expression levels of enzymes under mild drought and well‐watered conditions are indicated in dark and light red, respectively. Expression values are represented as values relative to the expression value at the leaf base in well‐watered plants. Values are average ± standard error (*n* = 3). CPS, ent‐copalyl diphosphate synthase; KS, ent‐kaurene synthase; KO, ent‐kaurene oxidase; KAO, ent‐kaurenoic acid oxidase.

The reason for the shift and reduction in the GA profile was examined by measuring all GA biosynthetic intermediates from GA_12_ onwards and two major GA inactivation products (GA_29_ and GA_8_; Figure 4). The levels of the first‐formed GA (GA_12_) were increased by the drought treatment, but the levels of the subsequent metabolites (GA_15_ and GA_24_) were significantly lowered. The GA biosynthetic intermediates GA_9_, GA_51_ and GA_34_ were hardly detectable, and differences in their levels were not significant. For most metabolite precursors of GA_4_ and GA_1_ (GA_15_, GA_44_, GA_24,_ GA_20_), a significant interaction between the drought treatment and the position along the growth zone was shown, indicating that the accumulation pattern of the GA metabolites was shifted towards the leaf base. The lower levels of bioactive GAs also resulted in a significant reduction in the levels of the *GA2‐OXIDASE* (*GA2‐OX*)‐mediated degradation products (GA_29_ and GA_8_; Figure 4).

The expression levels of several GA biosynthetic genes, as well as of the GA inactivation genes, *GA2‐ox*, were analysed by qRT‐PCR (Figure 4). No significant down‐regulation of the expression levels of *CPS* (ent‐copalyl diphosphate synthase), *KS* (ent‐kaurene synthase), *KO* (ent‐kaurene oxidase) or *KAO* (ent‐kaurenoic acid oxidase) was observed in the growth zone, whereas all tested genes encoding GA metabolic enzymes from the GA13 oxidation step onwards were significantly down‐regulated by the drought treatment, with the gene encoding the rate‐limiting biosynthetic enzyme GA20‐OXIDASE (Nelissen *et al*., 2012) being the earliest GA biosynthetic enzyme to show a lowered and shifted expression pattern.

### High GA levels in *GA20OX*‐overexpressing plants do not affect the response to mild drought

**Figure 5:**
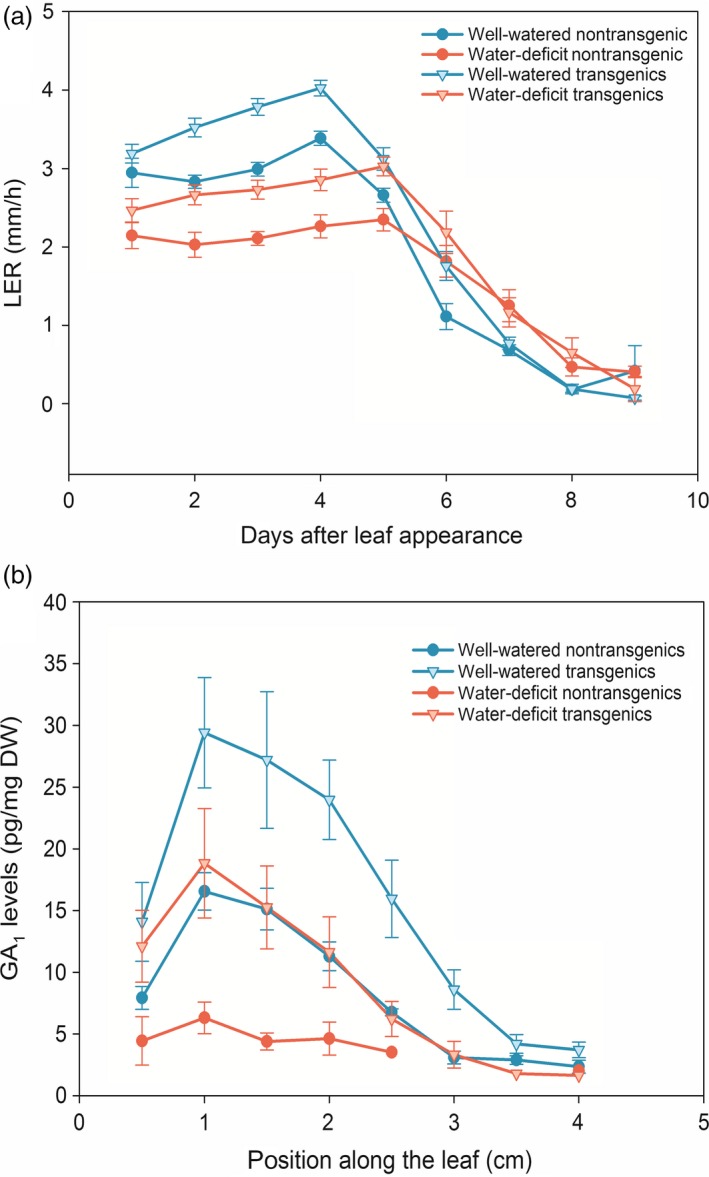
Growth curve (a) and GA1 levels (b) of hemizygous *GA20OX‐1* ^*OE*^ transgenic and nontransgenic siblings grown under well‐watered and water‐deficit conditions. (a) *n* ≥ 12, error bars represent standard error; (b) *n* = 3, error bars represent standard error.

Because mild drought lowered the expression level of *GA20‐OXIDASE*, boosting GA levels by overexpressing the *GA20‐OXIDASE* could render plants more tolerant to drought. To test this hypothesis, we grew hemizygous *GA20OX‐1* ^*OE*^ plants under mild drought conditions. Although under well‐watered conditions, leaf growth of the *GA20OX‐1* ^*OE*^ plants was enhanced compared with the nontransgenic siblings (Nelissen *et al*., 2012), the relative growth reduction caused by mild drought was comparable (26% and 23% in the hemizygous *GA20OX‐1* ^*OE*^ plants and the nontransgenic siblings, respectively; Figure 5a). The mild drought assay was repeated using homozygous *GA20OX‐1* ^*OE*^ plants and the nontransgenic siblings, showing similar results (23% and 24%, respectively). Kinematic analysis revealed that mild drought caused a significant reduction in the DZ size (24%) in the homozygous *GA20OX‐1* ^*OE*^ plants, which was similar to that observed for the nontransgenic siblings (22%), indicating that high levels of GA were not sufficient to overcome the reduction in the DZ size caused by mild drought (Table 2). The differences in the GA_1_ levels in the *GA20OX‐1* ^*OE*^ plants and their nontransgenic siblings under well‐watered and water‐deficit conditions were concomitant with the changes in the growth curve (Figure 5a). The water deficit caused a comparable reduction in GA_1_ levels for both transgenic and nontransgenic siblings, but the GA_1_ levels in the transgenic plants grown under drought were comparable or higher than those in the nontransgenic siblings grown in well‐watered conditions (Figure 5b).

**Table 2:** Growth‐related parameters of mild drought‐treated and well‐watered plants for the homozygous *GA20OX‐1* ^*OE*^ transgenic and nontransgenic siblings

Parameters	*GA20OX‐1* ^*OE*^ nontransgenic siblings				*GA20OX‐1* ^*OE*^ transgenic siblings			
	Control*	Drought*	% Change	*P*‐value#	Control*	Drought*	% Change	*P*‐value#
LER (mm/h)	3 ± 0.1	2.2 ± 0.1	−24	8.10^−6^	3.8 ± 0.3	2.9 ± 0.2	−23	0.001
FLL (mm)	560 ± 6.4	468.8 ± 13.2	−16	0.001	816.5 ± 14.6	669.5 ± 32.5	−18	0.09
Lma (μm)	119 ± 2	97 ± 2	−18	8.10^−4^	127 ± 2	98 ±4	−23	0.008
Lez (mm)	30.5 ± 3.1	25.5 ± 0.6	−16	0.2	44.3 ± 8.1	30.9 ± 5.3	−22	0.004
Nez	412 ± 41	403 ± 28	−2	0.9	504 ± 83	460 ± 81	−9	0.7
*P* (cells/h)	25 ± 0.3	23 ± 0.4	−7	0.03	30 ± 1	30 ± 1	−0.4	0.9
Ldz (mm)	14.8 ± 0.5	11.5 ± 0.3	−22	0.006	22.9 ± 0.5	17.5 ± 0.5	−24	0.005
Ndz (cells)	694 ± 32	540 ± 16	−22	0.025	1026 ± 67	795 ± 63	−22	0.048
*D* (cells/cells.h)	0.036 ± 0.002	0.043 ± 0.002	19	0.04	0.029 ± 0.003	0.038 ± 0.005	31	0.2
Tc (h)	19 ± 1	16 ± 1	−16	0.05	24 ± 2	19 ± 2	−22	0.1

^*^Mean ± standard error. ^#^ *P*‐values as obtained by Student's *t*‐test (*n* = 3).

LER, leaf elongation rate; FLL, final leaf length; Lma, mature cell length; Lez, expansion zone size; Nez, number of cells in expansion zone; *P*, cell proliferation rate; Ldz, division zone size; Ndz, number of dividing cells; *D*, cell division rate; Tc, cell cycle duration.

### Leaf Growth Viewer (LGV) as a tool to query high‐resolution transcriptomics data

Because mild drought might act through additional mechanisms besides GA biosynthesis to affect the position of TZ1, we compared high‐resolution, genome‐wide transcriptomics data obtained by sampling the growth zone with 0.5‐cm intervals till TZ2 of mild drought‐treated and *GA20OX‐1* ^*OE*^ plants (up to five and eight cm from the leaf base, respectively; Figure 1). The mild drought experiment was complemented with a sample representative of the late expansion zone (between 4 and 5 cm) and one of the MZ (between 8 and 9 cm; Figure 1). We developed an online search engine, called Leaf Growth Viewer (LGV; accessible through the following link: https://psblgv01.psb.ugent.be) that allows to query the data set starting from gene identifiers to obtain their leaf growth zone‐specific expression profile, or to search for genes that follow a specific expression profile.

The obtained expression profiles can be exported as image files (heat maps) or data list files. Because all gene identifiers are linked to PLAZA3.0 (Proost *et al*., 2015), each query results automatically and in parallel with the expression profiles in a gene ontology (GO) enrichment and shows the Arabidopsis homologues for the selected genes, which can also be easily exported as tabular data.

### Transcriptional changes within the division and expansion zone discriminate each zone into a basal and distal part

Because the two controls (well‐watered and nontransgenic *GA20OX‐1* ^*OE*^ plants) were both in the B104 background and all 0.5‐cm samples contained the same zones (Figure 1), their data sets were merged and statistically analysed together. 1020 genes were significantly up‐regulated along the leaf gradient (FC > 2; FDR < 0.05), of which 611 between the EZ and the MZ, and 1055 genes were significantly down‐regulated (FC < −2; FDR < 0.05), of which 661 between the EZ and the MZ (Table S1).

**Figure 6:**
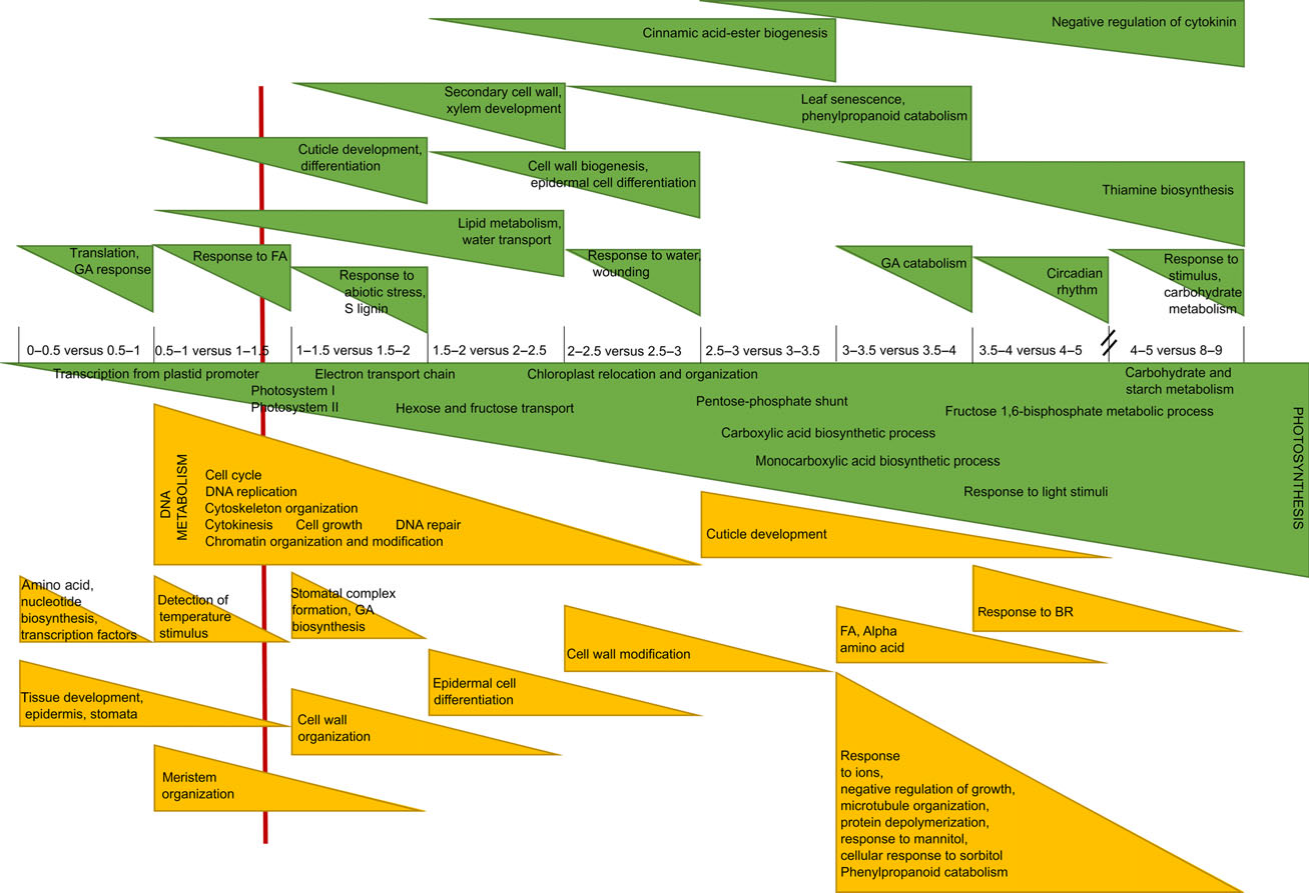
Schematic overview of the main categories of transcriptional changes between the sequential samples (Figure 1; 0–9 cm from the leaf base) in a growing maize leaf in well‐watered conditions. The GO categories of transcripts that were up‐regulated and therefore associated with the maturity of the tissue are indicated in green, and the GO categories of transcripts that were down‐regulated are indicated in yellow. The red vertical line indicates the transition zone between cell division and cell expansion (TZ1). GO enrichment is characterized by PLAZA.

Transcriptional changes were observed between the basal part and the more distal part of the DZ. The genes that were more highly expressed in the basal part were enriched in nucleotide and amino acid biosynthesis and transcriptional regulation (Figure 6; Table S1c). Ten transcription factors were specifically up‐regulated at the base of the DZ, among which four Dof (DNA‐binding with one finger) zinc finger transcription factors, GROWTH REGULATING FACTOR15, three TGACG SEQUENCE_SPECIFIC BINDING PROTEIN (TGA) transcription factors (among which FASCIATED EAR4 (FEA4) and LIGULELESS2), BEL1‐like homeodomain transcription factor and ETHYLENE REGULATED FACTOR1 (Table S1c). Conversely, the genes that were less expressed in the basal half cm compared with the more distal half cm of the DZ were involved in GA‐mediated signalling (gibberellin 3‐beta‐dioxygenase 2‐2 and GATA transcription factor 22) and translation (Figure 6; Table S1b).

Between the DZ and TZ1, genes with a function in ‘tissue development’, ‘epidermis’, ‘stomata’, ‘cell fate’, ‘growth’ and ‘maintenance of meristematic identity’ were down‐regulated. Towards the TZ1, the processes of ‘DNA replication’, ‘chromatin assembly’, ‘chromosome organization’, ‘cytokinesis’ and ‘cell cycle’ were enriched among the down‐regulated genes relative to the DZ samples (Figure 6; Table S1b). The down‐regulation of genes involved in ‘DNA metabolism’, ‘cell cycle’ and ‘cytokinesis’ persisted till after TZ1, whereas the process of ‘meristem organization’ was specifically down‐regulated at TZ1, and ‘GA biosynthesis’ (gibberellin 3‐beta‐dioxygenase 2‐2) dropped directly after TZ1. The sample containing TZ1 was also characterized by the up‐regulation of distinct transport pathways (oligopeptide, peptides, amide and water) and the GO categories ‘response to abiotic stimulus’, ‘light intensity’, ‘(far) red’, ‘UV‐A’, ‘fatty acids’, ‘blue light’ and ‘radiation’. In the early EZ samples, relative to the TZ1 sample, genes involved in ‘lignin biosynthesis’, ‘biogenesis of cinnamic esters’ and ‘secondary metabolism’ were up‐regulated, indicating that lignification starts soon after TZ1. In the later EZ, many genes involved in ‘phenylpropanoid metabolism’ and ‘cell wall organization’ were up‐regulated as compared with the early EZ samples. Towards the distal part of the EZ and TZ2, genes involved in ‘leaf senescence’ and ‘negative regulation of cytokinin’ and ‘GA catabolism’ were up‐regulated relative to the early EZ samples (Figure 6; Table S1c). Towards the distal EZ and the MZ, compared with the early EZ samples, genes involved in response to ions (magnesium, calcium and potassium) and mannitol and sorbitol were significantly down‐regulated, while genes involved in ‘thiamine biosynthesis’ were up‐regulated. In the MZ, ‘growth’, ‘protein polymerization’, ‘microtubule organization’ and ‘response to brassinosteroids’ were down‐regulated relative to the EZ.

Remarkably, the category ‘photosynthesis’ was significantly up‐regulated at all comparisons between successive samples from the leaf base to the MZ, starting within the DZ. A distinction could be made between different subprocesses of photosynthesis as cells progressed through the different zones. At the leaf base, genes involved in the biosynthesis of precursors of photosynthetic pigments were already expressed, and ‘transcription from plastid promoters’ was up‐regulated at the basal half cm compared with the next half cm. Around TZ1, an enrichment in the genes involved in light harvesting through photosystem I (PS I) and subsequently PS II was observed, relative to the DZ samples. The GO categories ‘chloroplast ribulose bisphosphate carboxylase complex biogenesis’ and ‘electron transport chain’ were up‐regulated from TZ1 to EZ, followed by ‘chloroplast relocation and organization’ between TZ1 and more basal EZ. Towards the end of the distal EZ, the GO categories ‘pentose‐phosphate shunt’ and ‘carboxylic acid biosynthetic process’ were up‐regulated relative to the early EZ. At TZ2, genes involved in the ‘circadian rhythm’ were significantly up‐regulated, while the genes involved in the actual production of sugars and starch were up‐regulated towards the MZ as compared with earlier samples (Figure 6; Table S1b and c).

### Mild drought stimulates a transcriptional reprogramming and lowers photosynthesis in the division zone and extends cell division at the transition zone

**Figure 7:**
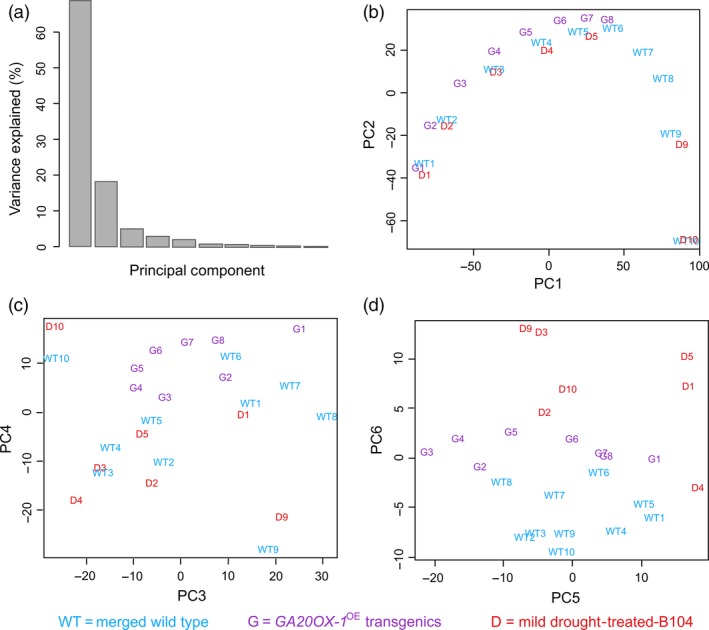
Principal component analysis (PCA) of the transcriptomics data. (a) Bar chart showing the explained variance for each component of PCA. (b–d) PCA 1, 2, 3, 4, 5 and 6 representing the classifications of the transcriptomics data. The numbers indicate the leaf samples: 1–10 as indicated in Figure 1 with mild drought (D), *GA20OX‐1* ^*OE*^ (G) and the merged well‐watered and wild‐type control samples (WT).

A principle component analysis (PCA) using all samples normalized relative to the wild‐type samples showed that the majority of the transcriptional changes could be explained by six principle components (Figure 7a). The genes that positively contributed to the first principle component (PC1), which separates the samples according to their position along the leaf, were enriched for the GO categories ‘photosynthesis’, ‘response to abiotic stimulus’, ‘very long‐chain fatty acid metabolic process’ and ‘secondary cell wall biogenesis’. The genes that negatively affected PC1 were enriched in ‘DNA replication’, ‘cell cycle’ and ‘nucleosome assembly and organization’. PC2 also divided the samples according to their position along the leaf, but was the highest for the samples in the basal EZ compared with the samples in the DZ and the MZ. The genes that positively contributed to PC2 were enriched for ‘lipid metabolic process’, ‘response to osmotic stress’, whereas the cells that negatively contributed were mainly involved in ‘leaf senescence’, ‘response to jasmonic acid’ and ‘response to oxidative stress’. Both PC1 and PC2 caused a shift in the mild drought, control and *GA20OX‐1* ^*OE*^ samples, a difference that was opposite between mild drought and *GA20OX‐1* ^*OE*^ (Figure 7b) and that corresponded to the microscopically determined position of the TZs.

The genes that contributed to PC4 that discriminated between the DZ, EZ and MZ samples were enriched for ‘proline transport’, ‘lipid’ and ‘phenylpropanoid metabolic process’ (Figure 7c). Remarkably, it was mainly PC6 that discriminated the drought samples from all well‐watered samples, indicating that the transcriptional changes over the gradient were much more prominent than the ones due to drought or *GA20OX‐1* ^*OE*^. The genes that contributed to PC6 in the same direction as the mild drought classification were enriched in a plethora of ‘responses to abiotic stimulus’ (including heat and reactive oxygen species), whereas the oppositely regulated genes were mainly involved in the ‘carbohydrate catabolic process’ (Figure 7d).

LGV can also be used to narrow down the number of genes identified, because of chained queries (a query within a query). In this way, we searched for genes that have opposite expression profiles in TZ1 in plants grown under drought conditions and in the *GA20OX‐1* ^*OE*^ line. However, because the position of TZ1 changes in opposite direction in the mild drought and *GA20OX‐1* ^*OE*^ samples (Figure 1), above comparison is likely to reflect mainly developmentally regulated genes. To resolve this, we performed a shift in both experiments, based on the cellular analyses that were used to determine the position of TZ1 (Table 1). As a consequence of the alignment of TZ1, all other zones shifted as well.

Few genes were specifically differentially expressed in *GA20OX‐1* ^*OE*^ plants (and not under mild drought) as compared with the wild type (16 or 1 genes at the DZ, 4 or 7 genes at TZ1 and 5 or 10 genes at the EZ, respectively; Table S2a and b).

In contrast, more genes, but still a modest set of genes, were specifically differentially expressed by mild drought (Table S2c and e): 140 and 76 at the DZ, 180 and 206 at TZ1 and 103 and 84 at the EZ, respectively. The genes that were up‐regulated by mild drought in the DZ were enriched for ‘regulation of gene expression’, ‘response to light intensity’, ‘response to reactive oxygen species’, ‘leaf morphogenesis’ and ‘stomatal complex formation’ (Table S2f). The genes involved in the ‘regulation of gene expression’ were several Dof transcription factors, two TGAs, ethylene response factor1 (ERF1), NAC67, SPEECHLESS, KNOTTED1 and a B3 domain‐containing transcription factor, of which some were also identified to be specific in the basal DZ (Table S1). Remarkably, at TZ1 and EZ, the GO categories ‘DNA replication’ and ‘cell cycle’ were significantly enriched among the genes that were up‐regulated by drought. These genes were mainly targets of E2F/DP because a significant enrichment was obtained of the TZ1 mild drought up‐regulated genes (31 of the 180) that showed homology to Arabidopsis genes that were computationally and experimentally shown to be E2F/DP targets (Table S3; Vandepoele *et al*., 2005; Verkest *et al*., 2014). At the EZ, several histones (H4, H2A, H2B) were significantly up‐regulated under mild drought, together with genes involved in DNA replication and cell cycle (Table S2e).

Among the drought‐mediated down‐regulated genes at the DZ are GA3‐oxidase (consistent with our Q‐PCR data, Figure 4), proline dehydrogenase involved in proline catabolism, and two transcription factors that were already associated with abiotic stress responses (Table S2c). Both at the DZ and TZ1, genes involved in PS II, RuBisCO (ribulose bisphosphate carboxylase) complex biogenesis and light harvesting complex were down‐regulated upon mild drought (Table S2d) and at TZ1 and EZ several aquaporins (PIP2‐3, PIP2‐4 and PIP2‐5) were significantly down‐regulated.

## Discussion

### High‐resolution sampling reveals the complex regulation of maize leaf growth

Kinematic analysis allows to quantify the contribution of cell division or cell expansion to a given leaf growth phenotype and to delineate zones in which cells are dividing (DZ) or expanding (EZ), until they reach their mature size (MZ; Nelissen *et al*., 2013, 2012). However, our data show that this rather simplistic growth model that merely discriminates between cell division, cell expansion and cell maturity is more complex. High‐resolution transcriptome analysis and quantification of hormones showed that within the DZ and EZ, there are substantial differences between the basal and distal parts of these zones. Indeed, the levels of auxin and tZ declined throughout the division zone, to reach their basal level around TZ1 (this work; Nelissen *et al*., 2012), reinforcing the concept that cells in the basal and distal parts of the DZ are molecularly distinct, whereas the presence of mitotic figures in the kinematic analysis (Nelissen *et al*., 2013) classified them all as dividing cells. These data suggest that there are gradients of molecular processes within the growth zone that govern cell cycle progression. It is likely that such gradients are at least partially mediated by diffusible factors, such as auxin, originating from the leaf base and gradually diminishing in concentration when cells are getting displaced further away from the leaf base.

This concept is also exemplified by a number of transcription factors that were specifically expressed in the basal half cm of the growing maize leaf, of which some were previously described to have a role in leaf development. The BEL1 domain‐containing protein, which is an orthologue of the Arabidopsis BELLRINGER, and FEA4, which is orthologous to Arabidopsis PERANTHIA, were both shown to regulate *AGAMOUS* (Bao *et al*., 2004; Pautler *et al*., 2015). The expression of *FEA4* during leaf growth coincides with the position where the highest levels of auxin were observed and FEA4 was shown to regulate many different transcription factors involved in leaf differentiation and polarity (Pautler *et al*., 2015). Other genes that are specifically highly expressed in the basal half cm are *LIGULELESS‐2*, which is involved in leaf patterning along the proximo‐distal axis (Walsh *et al*., 1998) and *GROWTH REGULATING FACTOR15* (*GRF15*). Previously, we have shown that *GRF15* was more highly expressed in the DZ compared with the EZ and that that GRF15 protein was identified as a significantly enriched part of the AN3‐mediated chromatin remodelling SWI/SNF complex in the DZ (Nelissen *et al*., 2015).

The fine sampling strategy also underlines the importance of TZ1 as an integration point of molecular cues because both at the transcript and at the hormone level, numerous changes occur exactly at TZ1. Previously, the local accumulation of GA at TZ1 resulted in a more distal shift of TZ1 and hence in more cells in the DZ (Nelissen *et al*., 2012). Here, we show that the more proximal shift of TZ1 under mild drought was associated with lowered levels of bioactive GA, which was also observed when analysing the biosynthetic intermediates and transcript levels of GA biosynthetic enzymes.

Some processes, such as photosynthesis, were found to be transcriptionally regulated in a gradient throughout the entire growth zone, although the high‐resolution sampling allows for discriminating subprocesses that show spatial specificity. At the leaf base, in the DZ, an up‐regulation was observed of genes enriched in the transcription from plastid promoters. Genes involved in plastid gene expression and the regulation of photosynthesis typically anticorrelate with final leaf size and timing of growth‐related parameters (Baute *et al*., 2015, 2016). Already at the leaf base, which is shielded from light by the surrounding older leaves, cells are preparing for their final role in photosynthesis, showing that the basal part of the growing leaf is instrumental to determine the final size and shape, as well as its role as energy source of the plant.

### Perturbations of the organization of the growth zone allow to identify novel mechanisms

Positively and negatively disturbing the organization of the growth zone allowed the identification of novel mechanisms. Remarkably, although the final level of the cytokinin iP was the same in all examined conditions, its level transiently increased in a zone corresponding to the TZ2, which shifted, together with TZ1, more basally or more distally, under mild drought and in *GA20OX‐1* ^*OE*^, respectively. As cytokinins have been shown to promote shoot development, to inhibit leaf senescence and to delay differentiation (Kieber and Schaller, 2014), it is unlikely that the increase in cytokinin is causing the transition between cell expansion and maturation. On the other hand, it is known that cytokinin influences chloroplast ultrastructure, number and biosynthesis (Cortleven and Schmülling, 2015). Because the increase in cytokinin occurs at a position that is close to the point of leaf emergence from the sheet and that thus might already perceive incident light, we hypothesize that towards the end of the EZ, cytokinin biosynthesis is activated to steer the development and activity of chloroplasts. This increase in cytokinin levels is concomitant with an increased expression in multiple successive samples in the EZ and MZ of a gene encoding an SCF‐type F‐box protein that is the orthologue of KISS ME DEADLY‐LIKE2 (KMD2). Elevated *KMD2* expression targets the B‐type Arabidopsis response regulators (ARRs), key transcription factors in the cytokinin response for degradation (Kim *et al*., 2013).

SA was found to gradually accumulate within the DZ, a phenomenon that became more apparent when the levels of SA were elevated under mild drought. An increase in endogenous levels of SA promotes stomatal closure and drought tolerance (Miura *et al*., 2013; Miura and Tada, 2014) and SA can influence both plant growth and drought tolerance in a dose‐dependent manner (Kang *et al*., 2012, 2013; Miura and Tada, 2014). Because stomata start to form within the DZ, the up‐regulation of SA in the DZ might reflect a role of SA in stomatal closure. The extended high levels of SA in the expanding and the mature part of the leaf might be more related to the antioxidant defence system of SA to protect against oxidative damage caused by drought (Miura and Tada, 2014).

Although ABA and GA have been reported to act antagonistically during germination, growth and flower development (Weiss and Ori, 2007; White *et al*., 2000), ABA was significantly up‐regulated in the *GA20OX‐1* ^*OE*^ transgenic plants, albeit to the same level as under mild drought. Because high levels of ABA inhibit GA biosynthesis (Toh *et al*., 2008), the elevated ABA levels in the *GA20OX‐1* ^*OE*^ plants might serve as a negative feedback loop to limit the maximal GA levels that can be obtained in the plants. Conversely, the drought‐evoked increase in ABA levels might result in an inhibition of GA biosynthesis, resulting in the lower levels of bioactive GA and its precursors under mild drought.

### Growth reduction by mild drought stress cannot be reversed by elevated GA levels

Because overexpression of the rate‐limiting enzyme GA20‐OXIDASE cannot render plants relatively more tolerant to mild drought, a comparative analysis was performed to identify transcripts that were differentially expressed specifically under mild drought and not in *GA20OX‐1* ^*OE*^ transgenic plants. Remarkably, five of the ten transcription factors that were highly expressed at the leaf base in normal conditions were significantly up‐regulated under mild drought. One of the up‐regulated *Dof* (*Dof22*) transcription factor genes has previously been shown to be up‐regulated in maize seedlings under salt treatment (Chen and Cao, 2015). The ERF1 protein is orthologous to RELATED TO APETALA2‐2 (RAP2.2), for which it has been shown that elevated levels sustain ABA‐mediated activation of stress response genes (Papdi *et al*., 2015). Also, the expression of *KNOTTED1* (*KN1*) is specifically up‐regulated by mild drought in the DZ. Interestingly, *KN1* has been shown to negatively modulate the accumulation of GA through the control of *GA2‐OXIDASE* and overexpression of *KN1* results in smaller plants (Bolduc and Hake, 2009), raising the possibility that the up‐regulated *KN1* expression might be involved in the drought‐mediated growth reduction.

Genes involved in proline accumulation, photosynthesis and aquaporins were specifically down‐regulated by drought stress. The down‐regulation of *PROLINE DEHYDROGENASE* (*ProDH*) during stress is widely accepted to promote proline accumulation (Verslues and Sharma, 2010). Our results are consistent with the down‐regulation of *ProDH* at the transition from cell division to cell expansion under mild drought in Arabidopsis (Clauw *et al*., 2015). Also, genes involved in PS II and the light harvesting complex were down‐regulated by mild drought at the DZ and TZ1, which is in line with the observation that drought stress results in damage to PS II and photochemistry in mature leaves (Lu and Zhang, 1999; Sperdouli and Moustakas, 2012). Transcriptomics data also revealed that several genes encoding PLASMA MEMBRANE INTRINSIC PROTEINS (PIPs), belonging to aquaporins that facilitate the water diffusion across cell membranes, were significantly down‐regulated at TZ1 and EZ by drought stress. Under water stress, the expression of several *PIPs* in leaves is down‐regulated, and ABA represses aquaporin activity (Alexandersson *et al*., 2005; Shatil‐Cohen *et al*., 2011). The down‐regulation of aquaporins might be a way for plants to minimize water flow through cell membranes and to maintain leaf turgor, which is required for both cell division and cell expansion (Chaumont and Tyerman, 2014).

Although increased GA levels could not compensate the mild drought‐induced growth reduction, the growth advantage of leaves from *GA20OX‐1* ^*OE*^ plants was maintained. The leaf length of *GA20OX‐1* ^*OE*^ plants under mild drought still exceeded that of the well‐watered nontransgenic siblings. These data indicate that the effects of growth‐promoting genes can still persist under mild abiotic stress conditions, although the genes are not necessarily involved in stress tolerance or survival.

### Plants grown under mild drought stress have adapted their growth, molecularly and cellularly

Our kinematic analysis showed that the cell division rate and the cell cycle duration were not significantly affected by mild drought, and also at the transcript level, no differential expression of cell cycle‐related genes was observed at the DZ. However, effects on cell division rate and down‐regulation of cell cycle genes have been observed in other studies that examined the effect of drought in the maize leaf (Avramova *et al*., 2017, 2015), indicating that the significance of differential expression of cell cycle genes might depend on the applied drought stress or additional environmental changes between different growth chambers. Alternatively, our high‐resolution analysis and the alignment of the samples according to the position of the TZ1 might explain some of the differences. By aligning the samples according to the TZ1, transcripts that are known downstream targets of E2F/DP transcription factors, which are well‐known negative regulators of the G1/S transition whose ectopic expression results in an increased cell division duration (De Veylder *et al*., 2002), were significantly up‐regulated at TZ1 and the basal EZ under mild drought during steady‐state growth. These data suggest that the mild drought‐induced expression of E2F/DP targets around TZ1 might provide the possibility to maintain the capacity to resume growth upon water availability.

During osmotic stress in Arabidopsis leaves, the initial reduction in cell division is in part compensated by meristemoid divisions, generating extra pavement cells while forming stomata (Bergmann and Sack, 2007; Geisler *et al*., 2000; Skirycz *et al*., 2011). Unlike Arabidopsis, where the meristemoid divisions contribute substantially to the final leaf size (Gonzalez *et al*., 2012), the polarized stomatal divisions in maize barely affect it (Larkin *et al*., 1997). Instead, our data show that the prolonged duration of leaf growth (or LED), at least partly, compensates for the mild drought‐induced growth reduction. More studies will be needed to examine the biological relevance of the prolonged LED as a compensation for the drought‐induced growth reduction, and to analyse whether a prolonged LED allows to resume growth upon re‐watering. Recently, we identified PLA1, a cytochrome P450 78A, as one of the key players involved in the prolonged LED under mild drought and cold stress (Sun *et al*., 2017).

At the molecular level, the effects of the mild drought treatment only explained approximately 1 % of the variation in the transcriptomics data, indicating that the changes over the leaf gradient explained the majority of the transcriptional variation rather than the drought treatment. Alternatively, the plants that were subjected to mild drought throughout their entire life most likely adapted their metabolism, resulting in limited differences at the molecular level to be observed as opposed to acute drought stress responses. The imposed stress most likely does not exceed the tolerance limitations but is mild enough to not cause permanent damage, but to rather evoke a new physiological homeostasis (Gaspar *et al*., 2002; Pinheiro and Chaves, 2011; Verslues *et al*., 2006).

### Leaf Growth Viewer allows querying transcriptome changes over the leaf growth gradient under mild drought stress and at elevated GA levels

To date, many different research groups use the maize leaf as a model, albeit with a different biological question (Bonhomme *et al*., 2012; Facette *et al*., 2013; Jaskiewicz *et al*., 2011; Li *et al*., 2010; Nelissen *et al*., 2012; Ponnala *et al*., 2014; Tausta *et al*., 2014; van Wijk *et al*., 2014; Zhang *et al*., 2014). We developed a user‐friendly and searchable tool, LGV, to visualize our transcriptomics data. LGV provides a scaffold that can be further developed to integrate multiple data sets and analytical tools. Recently, the AIM database in Arabidopsis (Wang *et al*., 2014) exemplified the need and power of integrating big data sets. Genes that are transcriptionally coordinated are often functionally related, indicating the importance to use different transcriptome studies to identify co‐expressed genes (De Bodt *et al*., 2010; Hansen *et al*., 2014). It has been shown that a comparative analysis of co‐expression networks over different species offers additional value to remove false positives and to increase the power of predictions (Hansen *et al*., 2014). In addition, proteins can interact with many different interactors and thereby often affect distinct processes (Tucker *et al*., 2001). Because more and more experimental data about proteome (Facette *et al*., 2013), metabolome (Wang *et al*., 2014) and protein–protein interactions (Bommert *et al*., 2013; Nelissen *et al*., 2015) become available in the maize leaf, together with the wealth of transcriptomics data (Li *et al*., 2010; Mattiello *et al*., 2014; Tausta *et al*., 2014; Wang *et al*., 2014), it would be a great opportunity to boost the maize leaf as a model system to understand organ growth in monocots by integrating these different levels of information. Such a multilevel integration, ultimately even complemented with phenotypes, could use LGV as a starting point.

## Experimental procedures

### Growth conditions

All experiments were executed in growth chambers with controlled relative humidity (55%), temperature (24 °C) and light intensity (170 mmol/m^2^/s photosynthetically active radiation at plant level) in a 16‐h/8‐h (day/night) cycle. For mild drought treatment, the water content was allowed to drop to a soil water content of 70% of the well‐watered condition, corresponding to a soil water potential of −0.518 and −0.023 MPa, respectively.

### Hormone profiling and microarray analysis

For hormone profiling, sampling, extraction, purification and hormone metabolic profiling were performed as described in Nelissen *et al*. (2012). The details of the microarray analysis are explained in Data S1.

### Leaf growth viewer

The back‐end of LGV is written in Python using the Django framework (https://www.djangoproject.com/), which connects to a MySQL database (https://www.mysql.com/). The dynamic HTML front‐end are JavaScript‐based, interactive charts using HighCharts (http://www.highcharts.com/).

## Conflicts of interest

No conflicts of interest are to be declared.

## Supporting information


**Table S1** The differentially expressed transcripts along the leaf gradient in the merged well‐watered and non‐transgenic *GA20OX‐1* ^*OE*^ control data set.

**Table S2** The specifically differentially expressed transcripts in *GA20OX‐1* ^*OE*^ transgenic plants under drought conditions.

**Table S3** List of E2F/DP targets that were specifically up‐regulated at the TZ1 by mild drought stress.

**Data S1** Details of the microarray analysis.
