# Supplementary material for: The reduction in maize leaf growth under mild drought affects the transition between cell division and cell expansion and cannot be restored by elevated gibberellic acid levels
Source: Plant Biotechnol J. 2017 Sep 4;16(2):615–27. doi: 10.1111/pbi.12801 (PMC5787831; doi:10.1111/pbi.12801)
Supplement: Supplementary file 4 — Data S1 Details of the microarray analysis. [file PBI-16-615-s003.docx]

**Supporting information**

**Microarray analysis**

The fourth leaf of maize B104 plants grown under well-watered or mild drought conditions was harvested two days after emergence from the sheath. The basal 2.5‑cm leaf samples together with the 4 to 5‑cm and 8 to 9‑cm leaf samples were used for transcriptome analysis. Five plants were sampled per biological replicate, and three biological replicates were taken for each genotype and each condition. Total RNA was isolated with the guanidinium thiocyanate-phenol-chloroform extraction method using TRI-reagent (Sigma-Aldrich). Total RNA was isolated with the guanidinium thiocyanate-phenol-chloroform extraction method using TRI-reagent (Sigma-Aldrich). Total RNA was hybridized in two loop designs for drought or *GA20OX-1^OE^* with dye swap on maize oligonucleotide two-color arrays printed by Maize Oligonucleotide Array Project Array (maizearray.org). The loop for the drought experiment was designed to directly compare the samples under well-watered and mild drought treatment, and the adjacent samples in well-watered and mild drought conditions, respectively. The loop for the *GA20OX-1^OE^* experiment was designed to directly compare the *GA20OX-1^OE^* samples with the non-transgenic leaf samples, and the adjacent samples in *GA20OX-1^OE^* transgenic and non-transgenic plants, respectively. Two biological replications of each loop were performed, and due to the color swap, this resulted in four values per comparison. Hybridizations were done according to the protocols provided by the maize array service. The mapping of the probes to the genes was done using the Maize Microarray Annotation database (http://MaizeArrayAnnot.bi.up.ac.za/). Probes were retained that only identified a single gene after BLAST (Coetzer *et al.*, 2011).

Background subtraction was performed using the normal-exponential model using a saddle-point approximation (Ritchie *et al.*, 2007; Silver *et al.*, 2009). Within-array normalization was done to correct for dye-bias with the Loess normalization method with default values for the span width (Smyth and Speed, 2003; Yang *et al.*, 2002; Yang *et al.*, 2001). Between-array normalization was done using the Aquantile method developed by Yang and Thorne (2003). The preprocessing steps were done at the probe level. Subsequently, the log2 expression values were averaged over probes targeting the same gene. After averaging, some filtering was performed: only probes with a log2 expression intensity higher than 10% of the 95th quantile of the log2 expression values of the negative control were retained. One outlying sample (WT1) was removed based on an MDSplot. A linear model was fitted to the log2 intensity values of each of these retained genes. All combinations of the two factor levels genotype and layer were converted to single factor levels to facilitate making the a priori group comparisons that motivated our study. Contrasts of interest were the identification of differentially expressed genes between subsequent layers in wild type, and within the same layer, between wild type and the treated condition and the mutant genotype. Moderated t-statistics were calculated with the empirical Bayes method (Smyth, 2004). P-values were adjusted for each contrast separately using the FDR method from Benjamini and Hochberg (1995). A gene was called differentially expressed when the adjusted P-value was smaller than 0.05 and the log fold change at least 1. All analyses have been done with the limma package for R (R Core Team, 2015; Ritchie *et al.*, 2015). The principle component analysis (PCA) plot on transformed count data was done in R using the ‘pca’ function.

**References**

Benjamini, Y. and Hochberg, Y. (1995) Controlling the false discovery rate - a practical and powerful approach to multiple testing. *Journal of the Royal Statistical Society Series B, Statistical Methodology* **57**, 289-300.

Coetzer, N., Myburg, A.A. and Berger, D.K. (2011) Maize microarray annotation database. *Plant Methods* **7**, 31.

R Core Team (2015) R: A language and environment for statistical computing. Foundation for Statistical Computing, Vienna, Austria (<http://www.R-project.org/)>.

Ritchie, M.E., Phipson, B., Wu, D., Hu, Y., Law, C.W., Shi, W. and Smyth, G.K. (2015) *limma* powers differential expression analyses for RNA-sequencing and microarray studies. *Nucleic Acids Res.* **43**, e47.

Ritchie, M.E., Silver, J., Oshlack, A., Holmes, M., Diyagama, D., Holloway, A. and Smyth, G.K. (2007) A comparison of background correction methods for two-colour microarrays. *Bioinformatics* **23**, 2700-2707.

Silver, J.D., Ritchie, M.E. and Smyth, G.K. (2009) Microarray background correction: maximum likelihood estimation for the normal-exponential convolution. *Biostatistics* **10**, 352-363.

Smyth, G.K. (2004) Linear models and empirical bayes methods for assessing differential expression in microarray experiments. *Stat. Appl. Genet. Mol. Biol.* **3**, Article 3.

Smyth, G.K. and Speed, T. (2003) Normalization of cDNA microarray data. *Methods* **31**, 265-273.

Yang, Y.H., Dudoit, S., Luu, P., Lin, D.M., Peng, V., Ngai, J. and Speed, T.P. (2002) Normalization for cDNA microarray data: a robust composite method addressing single and multiple slide systematic variation. *Nucleic Acids Res.* **30**, e15.

Yang, Y.H., Dudoit, S., Luu, P. and Speed, T.P. (2001) Normalization for cDNA microarry data. In: *Microarrays: Optical Technologies and Informatics* (Bittner, M.L., Chen, Y., Dorsel, A.N. and Dougherty, E.R. eds), pp. 141-152. Proceedings of SPIE.

Yang, Y.H. and Thorne, N.P. (2003) Normalization for two-color cDNA microarray data. *Lecture Notes - Monograph Series* **40**, 403-418.
